# Integrating Traditional and Conventional Mental Health Practices: A Cross-Cultural Study from Tunisia

**DOI:** 10.1192/j.eurpsy.2026.11646

**Published:** 2026-07-31

**Authors:** L. S. Chaibi, M. Mouldi, M. S. Majoul, M. Cheour, F. Fekih-Romdhane

**Affiliations:** 1 Faculty of Medecine of Tunis; 2 Psychiatry; 3 Ibn Omran, Razi Hospital, Tunis, Tunisia

## Abstract

**Introduction:**

The coexistence of conventional psychiatric services and traditional healing practices presents both challenges and opportunities in mental health care, particularly in culturally diverse settings like Tunisia.

**Objectives:**

This study examines the interplay between these systems, assessing whether traditional practices hinder or enhance mental health outcomes and exploring pathways for integration.

**Methods:**

A descriptive study was conducted with 143 participants (60% female, mean age 29.6 years). Data were collected on sociodemographic profiles, mental health service utilization, reliance on traditional healing, and barriers to accessing conventional care. Findings were contextualized using comparative data from regional and global studies.

**Results:**

A significant proportion of participants (25%) used traditional healing methods for mental health concerns, while 40% had consulted non-conventional therapists. Barriers to conventional care included financial constraints, logistical challenges, perceived inefficacy of biomedical treatments, and fear of side effects. Cultural beliefs, lack of awareness, and stigma further influenced help-seeking behaviors. In Tunisia, supernatural explanations for mental illness were prevalent, with 77% of relatives attributing schizophrenia to non-medical causes. Notably, 70% sought traditional help before psychiatric consultation, yet adherence to modern treatments remained unaffected.

**Conclusions:**

Traditional healing remains a vital, culturally-rooted component of mental health care in Tunisia. Rather than a barrier, it represents an opportunity for collaboration. Integrating traditional and conventional approaches through respectful partnership, task-sharing, and culturally-sensitive care can improve access, reduce stigma, and enhance treatment outcomes in diverse populations.

**Disclosure of Interest:**

None Declared

